# The impact of dietary neem leaf on the growth and biochemical traits of rabbits

**DOI:** 10.1038/s41598-025-06905-x

**Published:** 2025-06-25

**Authors:** Abdalla Ali, Ahmed Adawy, Zeinhom Ismaiel, Manal Hussein, Abdelraheim Attaai

**Affiliations:** 1 Department of Animal and Poultry Production, Faculty of Agriculture, South Valley University, Qena, 83523 Egypt; 2 Department of Poultry Production, Faculty of Agriculture, Sohag University, Sohag, 82524 Egypt; 3 Department of Cell and Tissues, Faculty of Veterinary Medicine, Assiut University, Assiut, 71526 Egypt; 4 Department of Anatomy and Embryology, Faculty of Veterinary Medicine, Assiut University, Assiut, 71526 Egypt; 5 Department of Anatomy and Histology, School of Veterinary Medicine, Badr University in Assiut, New Nasser City Assiut, Egypt

**Keywords:** Neem, Leaves, Growing, Rabbit, Additive, Hepatocytes, Glycobiology

## Abstract

Neem is a plant used both as food and in traditional medicine. Its many active components, such as Carotenoids, Saponins, Triterpenoids and Nimbidin, may render it a beneficial feed additive for rabbits. Healthy weaned rabbits from breed V-line (VL) were selected to examine the effect of neem (*Azadirachta indica*) on growth performance, carcass traits, morphology, and blood parameters responses. Thirty-two V-line rabbits (45 days old) were randomly assigned to four groups (*n* = 8 per group): a control group (G1) receiving a basal diet, and three treatment groups (G2, G3, G4) receiving the basal diet supplemented with 5%, 10%, and 15% neem leaf powder, respectively. Neem leaf supplementation had no significant effect on the rabbits’ growth performance, live body weight, carcass weight, lungs and abdominal fat, dressing percentage and liver. There was a significant (*P* < 0.05) increase in intestine length in G4. Nevertheless, the cecum considerably shrank (*P* < 0.05) in G3 and G4, which might have a more negative impact on growth performance. Certain biochemical measures (albumin, globulin, triglycerides, LDL, total protein, cholesterol, glucose, AST, and ALT) did not exhibit significant variations. However, a significant (*P* < 0.01) drop in blood urea occurred after the higher concentration. A significant (*P* < 0.05) rise in HDL after neem supplementation. Histologically, the liver showed signs of hepatotoxicity in the group supplemented with neem leaves, such as abnormal hepatocytes’ nuclear membranes, pyknotic nuclei, karyorrhexis and karyolysis. Additionally, the portal and central veins were congested, and a greater number of Kupffer cells were seen. In conclusion, the findings suggest that dietary neem leaf supplementation may have adverse effects on rabbit health and performance, particularly at higher concentrations.

## Introduction

The production of rabbits has increased recently in Egypt and other countries, mostly to meet the growing demand for fresh meat for human consumption and to provide extra income^1^. Additionally, rabbit meat is of high quality, high in protein, and low in calories and fat^2^. Diet can be enhanced by supplementation of variable elements, including plants or their extracts, with fewer side effects, and is environmentally friendly to manage infectious illnesses, such as thyme and fenugreek^3^selenium^4^ginger^5^ and anise, lemon and mint oils^6^. These substances can stimulate appetite, regulate digestion and metabolism, have anti-diarrheal properties, and stimulate hormonal and immune systems^7^.

Neem, *Azadirachta indica*, is a plant used in food and a traditional cure. It has been found that neem leaf extracts contain anti-inflammatory, anti-carcinogenic, anti-genotoxic, and immune-modulatory properties^8–10^. Because of the health advantages of plants, many studies have been done on them. Neem has many incredible medicinal benefits because it contains many active components like limonoids, azadirachtin, meliantriol, salannin, nimbin, nimbidin and deacetylazadirachtinol^11,12^. Moreover, Neem helps destroy harmful bacteria and enhances metabolism^13–15^. According to Amin and colleagues in rats^8^ and Abu-Elala and colleagues in fish^16^their benefits are brought about by an increase in antioxidant defences and free radical scavenging. Neem trees are abundant. Using neem as a feed additive could benefit as an alternative feed and for its bioactive components. However, using neem is controversial among different researchers^11,12^. The current study aimed to investigate the possibility of using crude neem leaves, the simplest and cheapest to rabbit farmers, as a feed additive for rabbits, its adverse effects and the best dose.

## Materials and methods

### Experimental animals and management

All methods were performed following the relevant guidelines and regulations. All procedures of the current study were reported following ARRIVE guidelines. All procedures were conducted following the Animal and Poultry Production Department Ethical Committee, Faculty of Agriculture, South Valley University, Qena, Egypt, and were approved by the Ethics Committee (SVU-AGRI-91-2-2020), which ensured that the rabbits were treated throughout the experiment following the standards for the care of experimental animals, and every effort was made to minimize the suffering of the involved animals. All procedures were followed by veterinarians.

Unsexed growing V-Line rabbits of 36 days old, were obtained from a commercial local source. Rabbits were reared individually in standard-sized galvanized wire cages (width × length × height: 60 cm x 60 cm ×40 cm), equipped with an automatic drinker and a manual feeder. The animals were housed in climate-controlled rabbitry, with consistent sanitation and management practices. The ambient temperature was maintained at 22 to 26 °C and relative humidity from 20 to 35%. Fresh tap water was available Ad libitum via stainless steel nipples located inside each cage. Lighting was provided for rabbits for 16 h. The rabbits were kept for 3 days for adaptation before the experiment started.

## Experimental design

A total number of thirty-two rabbits were randomly divided into four groups. Each group involved eight rabbits, and each rabbit was considered a replicate. The first group (G1) was used as a control and fed on basal diets without neem supplementation. The second, third and fourth groups (G2, G3, G4) were fed basal diets supplemented with 5, 10 and 15% dry powder of neem leaves, respectively. The neem-supplemented feed was prepared weekly and mixed thoroughly. The neem leave powder was introduced gradually to the standard diet, starting at 2.5% on the first day, and increased daily by 2.5% till reaching the dose of 10% on the 4th day for G3 and 15% on the sixth day for G4. Their age was 45 days with a mean weight of 980 g at the start of the experiment after the adaptation period to the place and the experimental diet. During the total experimental period of 10 weeks, rabbits were housed under the same managerial, hygienic and environmental conditions. The ingredients and chemical composition of the standard diet of growing rabbits are presented in Table (1).

**Table 1:** Ingredients and chemical composition of the standard diet of rabbits.

Item	Diet		
Ingredients (%)			
Yellow corn	32.0	Vitamins and minerals premix^1^	0.3
Wheat bran	20.0	DL-Methionine	0.1
Soybean meal (44% CP)	18.0		
Wheat straw	12.0	**Chemical composition (%)**	
Alfalfa hay	5.0	Dry matter	91.4
Rice bran	5.0	Ash	9.8
Linseed straw	2.8	Crude protein	17.0
Sunflower meal	2.5	Ether extract	2.9
Lime stone	2.0	Crude Fiber	12.3
Sodium chloride	0.3	Gross energy (MJ kg^−1^)	18.2

1Vitamin and mineral premix, per kg of diet: vitamin A 10.000 IU, vitamin D 3.900 IU, vitamin E 50.0 mg, vitamin K 2.0 mg, vitamin B_1_ 2.0 mg, folic acid 5.0 mg, pantothenic acid 20.0 mg, vitamin B_6_ 2.0 mg, choline 1200 mg, vitamin B_12_ 0.01 mg, niacin 50 mg, biotin 0.2 mg, Cu 0.1 mg, Fe 75.0 mg, Mn 8.5 mg, Zn 70 mg.

Individual feed intake was recorded weekly at 8:00 am. Feed intake was calculated by subtracting the remaining feed from the total consumed feed. The feed containing neem leaves was introduced gradually to rabbits for 7 days, and then all rabbits were fed diets corresponding to their treatments. Body weight was recorded on the first day and every 14 days.

### Collection and preparation of neem leaves

Fresh neem leaves were harvested from neem trees around the experimental site at the poultry farm, Faculty of Agriculture, South Valley University, Qena. Then the leaves were dried in the open clean concrete floor space. The sun-dried leaves were milled using a commercial milling machine into neem leaf meal (NLM) according to the procedure described by^17^. Then the rabbits’ diets were ground, and the neem leaf powder was mixed with it and reshaped into pellets.

### Gas chromatography-mass spectrometry (GC-MS) analysis

The chemical composition of samples was analyzed using GC-TSQ mass spectrometer (Thermo Scientific, Austin, TX, USA) with a direct capillary column TG–5MS (30 m x 0.25 mm x 0.25 μm film thickness). The column oven temperature was initially held at 60 °C and then increased by 5 °C/min to 250 °C, withheld for 2 min, and then increased to 300 °C for 30 min. The injector temperature was kept at 270 °C. Helium was used as a carrier gas at a constant flow rate of 1 ml/min. The solvent delay was 4 min, and diluted samples of 1 µl were injected automatically using Autosampler AS3000 coupled with GC in the split mode. EI mass spectra were collected at 70 eV ionization voltages over the range of m/z 50–650 in full scan mode. The ion source and transfer line were set at 200 °C and 280 °C, respectively. The components were identified by comparison of their mass spectra with those of WILEY 09 and NIST14 mass spectral databases.

### Blood collection

At the end of the experiment, blood was drawn from the ear veins of 8 rabbits per treatment, using sterile needles and syringes to obtain serum. The blood was centrifuged for 10 min at room temperature (3000 g). Before being examined, the serum was collected in tubes and stored at −20 °C. Diagnostic kits (Biodiagnostics, Cairo, Egypt) were used to perform colorimetric measurement of liver enzymes, including aspartate aminotransferase and alanine transaminase, as well as kidney function tests like urea and creatinine (Sunostik Medical Technology Co., Ltd, China).

Blood samples were kept in dry clean centrifuge tubes without anti-coagulate and allowed to clot at 5^o^C for 2 h. After the serum was separated naturally, then centrifuged at 3000 rpm for 15 min. Blood serum was then obtained and stored at −20 °C for further analysis in a spectrophotometer. Then, analyses were conducted to determine the physiological responses. Total protein concentration, albumin, globulin, total cholesterol, triglyceride, high-density lipoprotein (HDL), low-density lipoprotein (LDL), glucose, aspartate aminotransferase (AST) and alanine aminotransferase (ALT) were measured.

### Carcass measurements

On the termination of the experimental period at 105 days of age, the rabbits were anaesthetized using intravenous injection of ketamine and xylazine. After 15 min, and when signs of anaesthesia were evident, by loss of the palpebral and pedal reflex, the rabbits were decapitated/slaughtered. The slaughtered rabbits bled, and then the skin, genitals, head, urinary bladder, gastrointestinal tract and the distal part of legs were removed. The head and full gastrointestinal tract were weighed and expressed as a percentage of the slaughter weight. Also, the length of the gastrointestinal tract was measured. The liver, pancreas, abdominal fat, spleen and heart were weighed.

### Histological examination

#### Sample collection and paraffin embedding

The tissue samples from the liver were collected from the slaughtered rabbits and fixed in a 10% neutral buffered formalin solution for 24 h. Tissues were dehydrated by dipping through a series of ethyl alcohols of increasing concentrations (from 70% to absolute), and cleared with xylene. The samples were then paraffin-embedded, and 5-micron thick sections were cut and stained with hematoxylin-eosin^18^for general examination.

#### Histochemical staining

Periodic acid-Schiff (PAS) reaction was used for the demonstration of glycogen content in hepatocytes. Picrosirius red and Crossman’s trichrome were used to demonstrate the fibrous tissue and the collagen content within the liver tissue^18^. The slides were examined using a light microscope (Olympus CX41) equipped with a digital Olympus camera (C-5060, Japan).

### Statistical analysis

The collected data was statistically analyzed by the One-Way Analysis Of Variance with the General Linear Model (GLM) procedure of the SAS Institute^19^. All statements of significance are based on the 0.05 level of probability. Significant differences among treatments were performed using Duncan’s multiple-range test^20^.

## Results

**Fig. 1:**
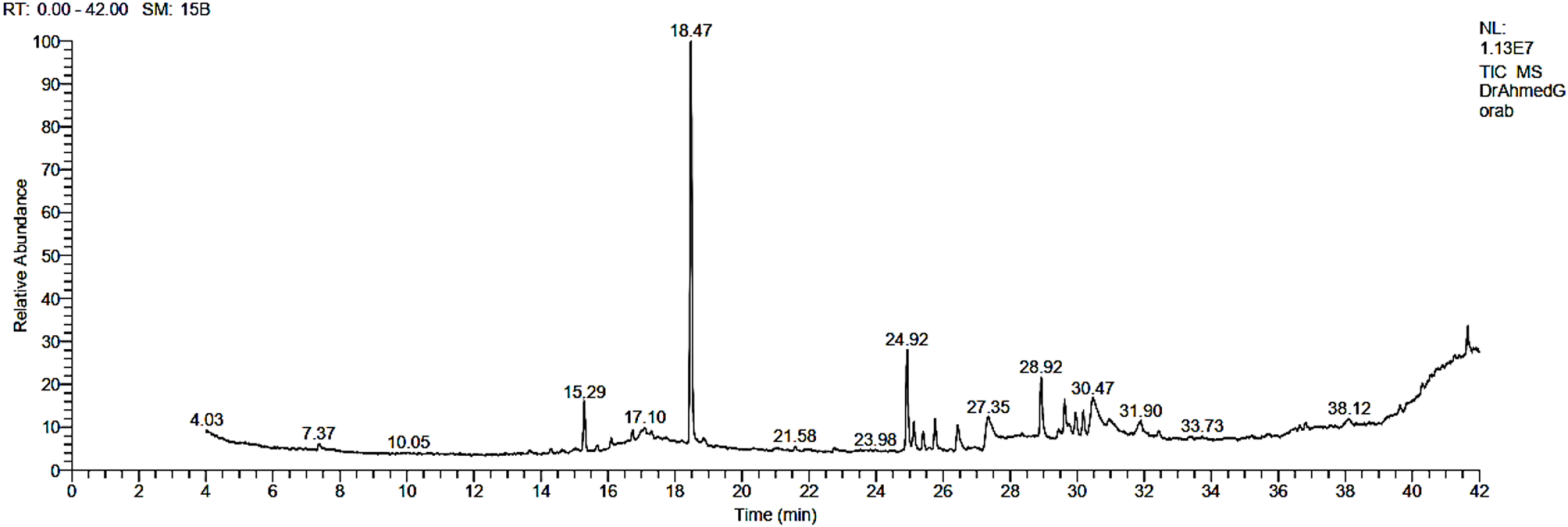
The gas chromatography-mass spectrometry (GC-MS) analysis for neem leaves.

The main components of the analyzed neem leaves by Gas chromatography-mass spectrometry (GC-MS) are summarized in Fig. (1) and Table (2).

**Table 2:** The main components of the Neem leaves analyzed by the gas chromatography-mass spectrometry (GC-MS).

RT	Compound Name	Area %	MF	Molecular Formula	M Wt	Cas #	Library
18.47	GERMACRENE B	28.22	927	C15H24	204	15423-5 7 − 1	WileyRegi stry8e
30.46	CHOLESTAN-3-OL, 2-METHYLENE-, (3á,5à)-	7.94	786	C28H48O	400	22599-9 6–8	WileyRegi stry8e
24.92	Neophytadiene	7.77	916	C20H38	278	504 − 96 −1	mainlib
27.34	9-OCTADECENOIC ACID (Z)-	7.2	889	C18H34O2	282	112 − 80 −1	WileyRegi stry8e
28.92	HEXADECANOIC ACID, TRIMETHYLSILYL ESTER	5.62	843	C19H40O2Si	328	55520-8 9 − 3	WileyRegi stry8e
15.29	BICYCLO[7.2.0]UNDEC-4-ENE, 4,11,11-TRIMETHYL-8-METHYLE NE-, [1R-(1R*,4E,9 S*)]-	3.69	933	C15H24	204	87-44-5	WileyRegi stry8e
26.43	Hexadecanoic acid, methyl ester	3.2	801	C17H34O2	270	112 − 39 −0	replib
29.63	9-OCTADECENOIC ACID (Z)-, METHYL ESTER	3.17	851	C19H36O2	296	112 − 62 −9	WileyRegi stry8e
31.9	Octadecanoic acid, 9,10-epoxy-18-(trimethylsiloxy)-, methyl ester, cis-	2.71	739	C22H44O4Si	400	22032-7 8 − 6	mainlib
25.76	3,7,11,15-Tetramethyl-2-hexadecen-1 -ol	2.42	893	C20H40O	296	102,608 −53-7	mainlib
25.12	3-Trifluoroacetoxydodecane	2.16	824	14H25F3O2	282	NA	mainlib
29.95	1,3,5-TRIAZINE-2,4-DIAMINE, 6-CHLORO-N-ETHYL-	2.11	785	C5H8ClN5	173	1007-2 8–9	WileyRegi stry8e

### Growth performance

The supplementation of neem leaves at 50, 100 and 150 gm/kg did not affect the body weight of rabbits during 45, 75 and 105 days of age **(**Table 3**)**. In addition, the supplementation of neem leaves at 50, 100, and 150 gm/kg to rabbits’ diets did not affect the body weight gain (BWG), feed intake and feed conversion ratio (FCR) during the experimental period compared to the control group.

**Table 3:** Effects of Neem leaves on growth performance of growing rabbits.

Items	Body weight, g/age			Body weight gain, g				Feed intake, g			Feed conversion ratio		
	45 d	75 d	105 d	1-30d		30–60 d	1–60 d	1-30d	30–60 d	1–60 d	1-30d	30–60 d	1–60 d
**Control**	1056 ± 66.95	2149.5 ± 109.75	3062.5 ± 116.34	1093.5 ± 88.26		913.0 ± 39.92	2006.5 ± 129.02	2810.5 ± 47.57	3673.0 ± 35	6483.5 ± 46.26	2.57 ± 0.08	4.02 ± 0.21	3.23 ± 0.11
**5% NL**	971.0 ± 136.62	1949.0 ± 155.29	2823.0 ± 105.57	978.0 ± 248.92		874.0 ± 80.04	1852.0 ± 321.41	2806.4 ± 21.57	3657.4 ± 34.26	6463.8 ± 36.87	2.86 ± 0.32	4.18 ± 0.41	3.49 ± 0.24
**10% NL**	916.2 ± 125.22	1853.0 ± 181.43	2657.6 ± 169.6	936.8 ± 171.72		804.6 ± 39.26	1741.4 ± 165.77	2671.8 ± 49.58	3646.6 ± 35.56	6318.4 ± 72.59	2.85 ± 0.24	4.53 ± 0.24	3.62 ± 0.15
**15% NL**	1033 ± 114.42	1983.3 ± 187.82	2823.3 ± 216.84	950.3 ± 299.91		840.0 ± 107.59	1790.3 ± 190.42	2728.7 ± 28	3598.3 ± 42.06	6327.0 ± 47.08	2.87 ± 0.35	4.42 ± 0.46	3.53 ± 0.23
***P***. **value**	0.843	0.639	0.324	0.659		0.689	0.376	0.070	0.623	0.109	0.672	0.753	0.484

### Carcass characteristic

**Fig. 2:**
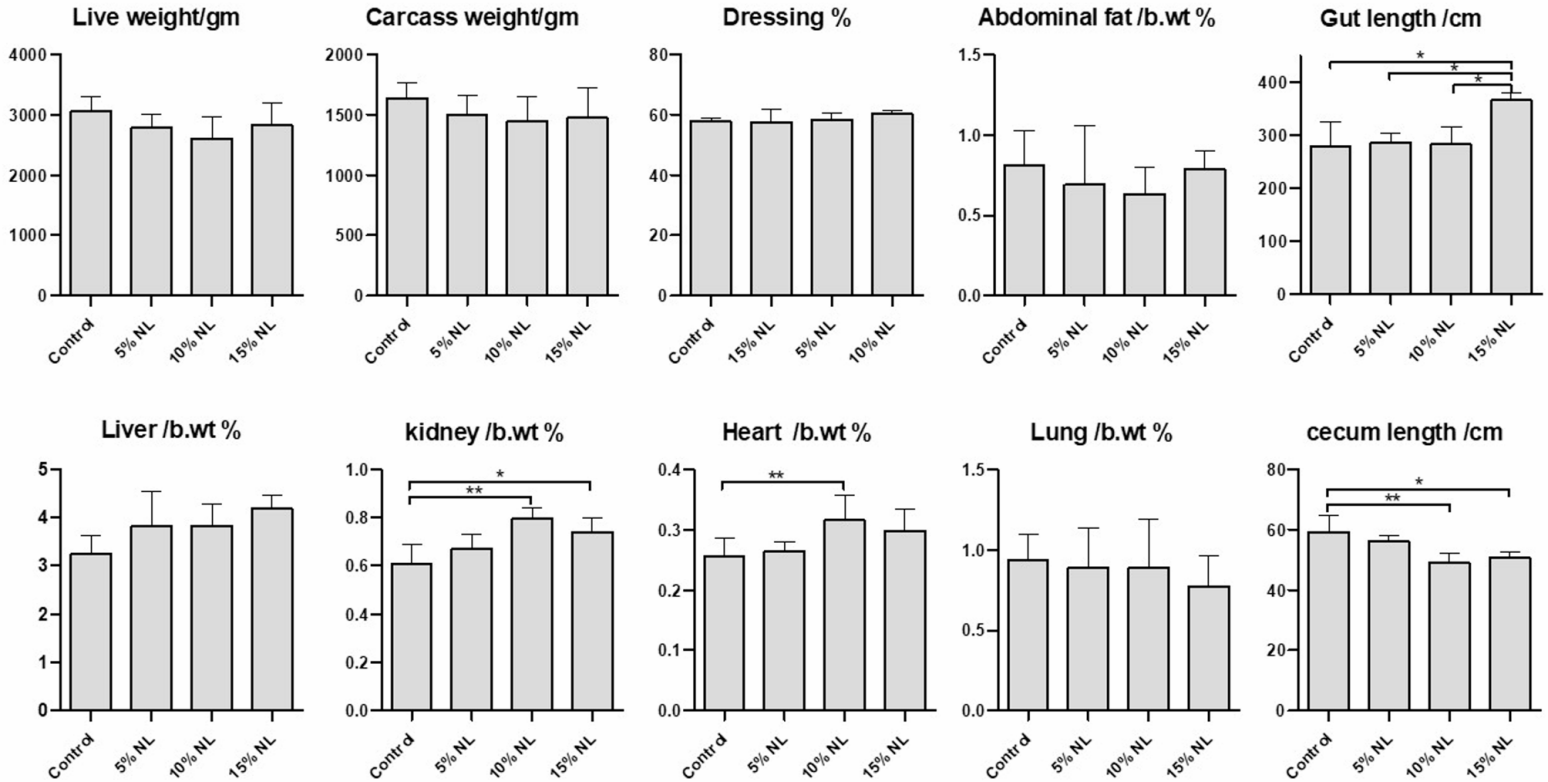
Carcass criteria for growing rabbits fed on neem leaves.

The supplementation of neem leaves at 50, 100 and 150 gm/kg to rabbits’ diets resulted in a significant increase (*P* < 0.05) in rabbits’ kidneys compared with the control group **(**Table 4; Fig. 2**)**. The heart was significantly increased (*P* < 0.05) with supplementation of neem leaves at 100 gm/kg to rabbits’ diets. In addition, the gut length significantly increased (*P* < 0.05) with supplementation of neem leaves at 150 gm/kg to rabbits’ diets. However, the cecum significantly decreased (*P* < 0.05) with supplementation of neem leaves at 100 and 150 gm/kg to rabbits’ diets. In contrast, the three concentrations did not affect the live body weight, carcass weight, dressing percentage, abdomen fat, lungs and liver, compared with the control group.

### Blood biochemical parameters

The blood biochemical parameters did not show any significant differences in terms of some parameters (total protein, albumin, globulin, triglyceride and LDL) with the supplementation of neem leaves at 50, 100 and 150 gm/kg to rabbits’ diets **(**Table 5**)**.

**Table 4:** Effect of Neem leaves levels on carcass criteria for growing rabbits.

Items	Control	5% NL	10% NL	15% NL	*P*.value
**Live weight**	3062.5 ± 116.34	2789.0 ± 100.70	2603.0 ± 162.92	2823.30 ± 216.84	0.207
**Carcass weight**	1637.5 ± 65.75	1499.0 ± 74.38	1443.0 ± 91.28	1476.70 ± 144.38	0.480
**Dressing %**	57.785 ± 0.05	58.4 ± 2.581	60.216 ± 0.6	57.523 ± 1.041	0.361
**Liver**	3.253 ± 0.18	3.8 ± 0.169	3.8368 ± 0.2	4.181 ± 0.32	0.160
**Kidney**	0.608^b^ ± 0.08	0.6^ab^ ± 0.063	0.77540^a^ ± 0.31	0.738^a^ ± 0.249	0.030
**Heart**	0.254^b^ ± 0.008	0.27^ab^ ± 0.021	0.31540^a^ ± 0.019	0.298^ab^ ± 0.021	0.050
**Abdomen Fat**	0.812 ± 0.108	0.68 ± 0.065	0.6334 ± 0.073	0.786 ± 0.166	0.709
**Lungs**	0.933 ± 0.080	0.887 ± 0.11	0.8888 ± 0.137	0.772 ± 0.111	0.851
**Cecum**	59.5^a^ ± 3.013	56.0^ab^ ± 2.08	49.0^b^ ± 0.969	50.8^b^ ± 2.081	0.025
**Gut length**	278.25^b^ ± 23.97	286^b^ ± 11.55	283^b^ ± 13.56	366^a^ ± 9.138	0.009

NL=neem leaves; SEM=the standard error of means.

In addition, the blood biochemical parameters did not show significant differences in some parameters (Cholesterol, glucose, creatinine, serum AST and ALT) with the supplementation of neem leaves at 50, 100 and 150 gm/kg to rabbits’ diets **(**Table 6**)**. However, the blood urea showed a significant decrease (*P* < 0.01) with the supplementation of neem leaves at 150 gm/kg to rabbits’ diets. The blood creatinine showed a significant decrease (*P* < 0.01) at 100 gm/kg and a significant increase (*P* < 0.01) at 150 gm/kg in rabbits’ diets with the supplementation of neem leaves. The HDL showed a significant increase (*P* < 0.05) with the supplementation of neem leaves in rabbits’ diets.

**Table 5:** The blood biochemical parameters after the addition of Neem leaves (NL) into growing rabbits’ diets.

Items	TP (mg/dl)	Alb (mg/dl)	GLOB (mg/dl)	TG (mg/dl)	HDL (mg/dl)	LDL (mg/dl)
**Control**	5.96 ± 0.33	3.50 ± 0.18	2.46 ± 0.15	198.8 ± 1.2	32.3^b^ ± 1.45	114.69 ± 2.64
**5% NL**	5.83 ± 0.26	3.93 ± 0.28	1.89 ± 0.52	201.2 ± 1.83	40.3 ^a^ ± 0.333	123.12 ± 8.8
**10% NL**	6.16 ± 0.22	3.85 ± 0.04	2.31 ± 0.24	209.9 ± 5.6	39.6^a^ ± 1.2	114.27 ± 5.23
**15% NL**	5.79 ± 0.2	3.93 ± 0.18	1.85 ± 0.38	203.6 ± 0.69	36.0^ab^ ± 2.08	114.64 ± 2.83
***P***.**value**	0.750	0.390	0.560	0.130	0.013	0.620

Serum total protein (TP), albumin (ALB), globulin (GLOB), triglyceride (TG), high-density lipoprotein (HDL), and low-density lipoprotein (LDL). a, b means with different superscripts on the same column are significantly different (P < 0.05). SEM is the standard error of means.

**Table 6:** The blood metabolic parameters after the addition of Neem leaves (NL) into growing rabbits’ diets.

Items	Cholesterol (mg/dl)	Glucose (mg/dl)	Urea (mg/dl)	Creatinine (mg/dl)	AST (mg/dl)	ALT (mg/dl)
**Control**	160.9 ± 2.8	89.3 ± 2.9	46.8^a^ ± 2.5	1.8^ab^ ± 0.03	25.06 ± 1.95	17.72 ± 3.62
**5% NL**	171.4 ± 8.61	92.9 ± 1.45	47.2^a^ ± 1.01	1.6^bc^ ± 0.02	31.12 ± 1.88	24.35 ± 6.07
**10% NL**	164.2 ± 4.77	90.4 ± 4.65	50.7^a^ ± 2.2	1.5^c^ ± 0.04	29.32 ± 9.8	17.80 ± 4.21
**15% NL**	162.5 ± 3.12	93.6 ± 2.62	35.6^b^ ± 0.24	1.9^a^ ± 0.09	32.78 ± 6.98	21.82 ± 8.16
***P***.**value**	0.553	0.740	0.001	0.009	0.817	0.81

Serum cholesterol, glucose, urea, creatinine, serum aspartate aminotransferase (AST), and alanine aminotransferase (ALT) as affected by the addition of neem leaves (NL) into growing rabbits’ diets. ^a, b^ means with different superscripts on the same column are significantly different (*P* < 0.05). SEM is the standard error of means.

### Morphological results

**Fig. 3:**
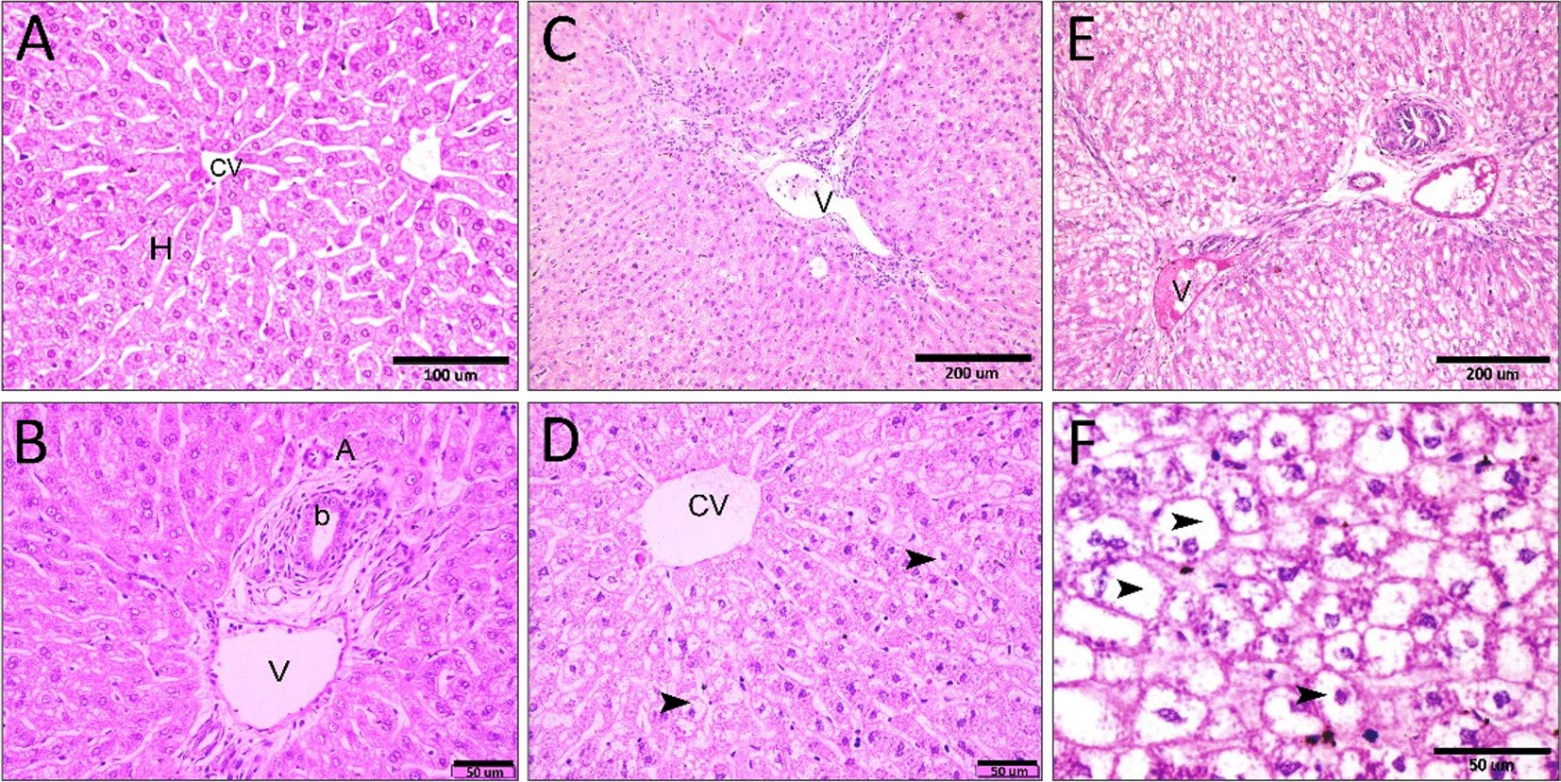
Liver histology after administration of Neem leaves. **(A)** the classical hepatic lobules formed of a central vein **(c)** with cords of hepatocytes **(H)** radiating from the central vein towards the periphery of the lobule. **(B)** The portal triad contained branches of the hepatic artery **(A)**, portal vein **(V)** and bile duct **(B)**. **(C-F)** Abnormal liver with congested portal vein, central vein, vacuolar degeneration (arrowheads) and diffuse and periportal leucocytic infiltration. PA portal area, H hepatocytes, V portal vein, CV central vein, A portal artery, b bile duct.

The liver plays an important role in the metabolism of ingested feed and other substances. Microscopically, the examination of Hx&E- stained sections of the liver collected from the control group (negative control group) which fed on a basal diet revealed the normal histological structure of the hepatic lobule (Fig. 3A-B). The control liver was formed of the classical hepatic lobules. Each lobule was formed of a central vein, with cords of hepatocytes radiating from the central vein towards the periphery of the lobule (Fig. 3A). Hepatocytes appear as large polygonal cells with round vesicular nuclei. The hepatic sinusoids are irregularly dilated vessels in intimate contact with the flanking hepatocytes. These sinusoids are lined by flat endothelial cells (Fig. 3A). The portal tracts appeared normal and contained branches of the hepatic artery, portal vein, and bile duct (Fig. 3B).

Rabbits fed on the neem leaves with different concentrations showed vacuolar degeneration. Moreover, both the central vein and portal vein were dilated and filled with blood (Fig. 3C-F). In addition, activation of Kupffer cells, which appeared with higher numbers, was observed. There are also diffused and periportal leucocytic infiltration (Fig. 3D-F). Notably, the hepatocytes in the group that received a higher concentration of leaves showed pyknotic nuclei, karyorrhexis and karyolysis with irregular nuclear membranes (Fig. 3E-F).

**Fig. 4:**
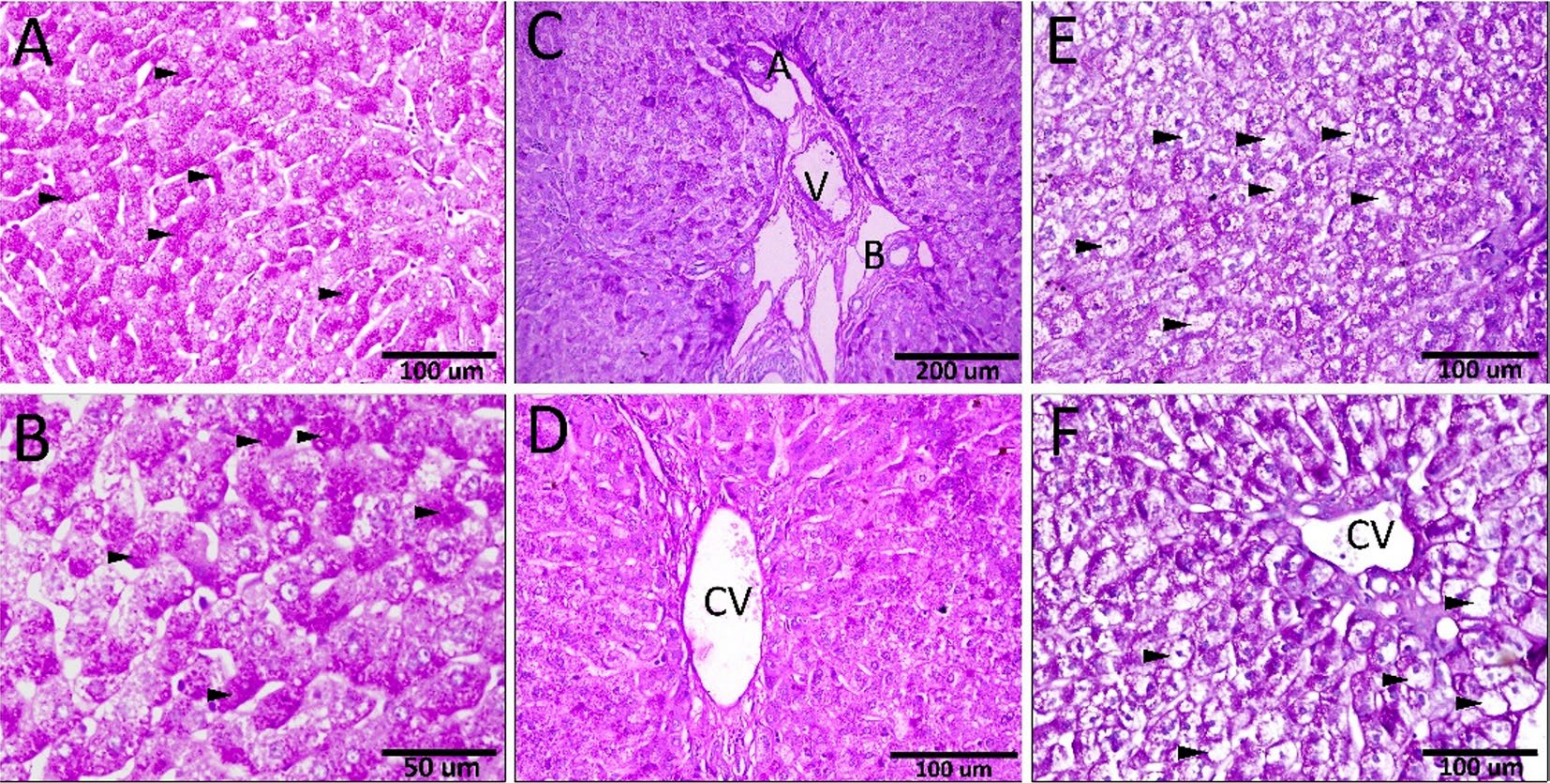
The glycogen content in the hepatocytes. (**A, B**) A strong magenta-stained granules (arrowheads) with PAS staining in the control groups. (**C, D**) The rabbits fed on leaves with a 50 gm/kg showed mild depletion of PAS + ve glycogen granules and (**E, F**) higher concentrations (100 & 150 gm/kg) showed severe depletion of glycogen content (arrowheads).

The glycogen content in the hepatocytes was demonstrated, utilizing the Periodic Acid-Schiff (PAS) reaction. The hepatocytes’ cytoplasm of the control group contained a high content of glycogen granules, which appeared as strong magenta staining with PAS staining (Fig. 4A, B). The rabbits fed on leaves with low concentration (5 gm/kg) showed depletion of PAS-positive glycogen granules in most hepatic lobules (Fig. 4C, D). Moreover, the rabbits fed on leaves with higher concentrations (10 & 15 gm/kg) showed severe depletion of glycogen content (Fig. 4E, F).

**Fig. 5:**
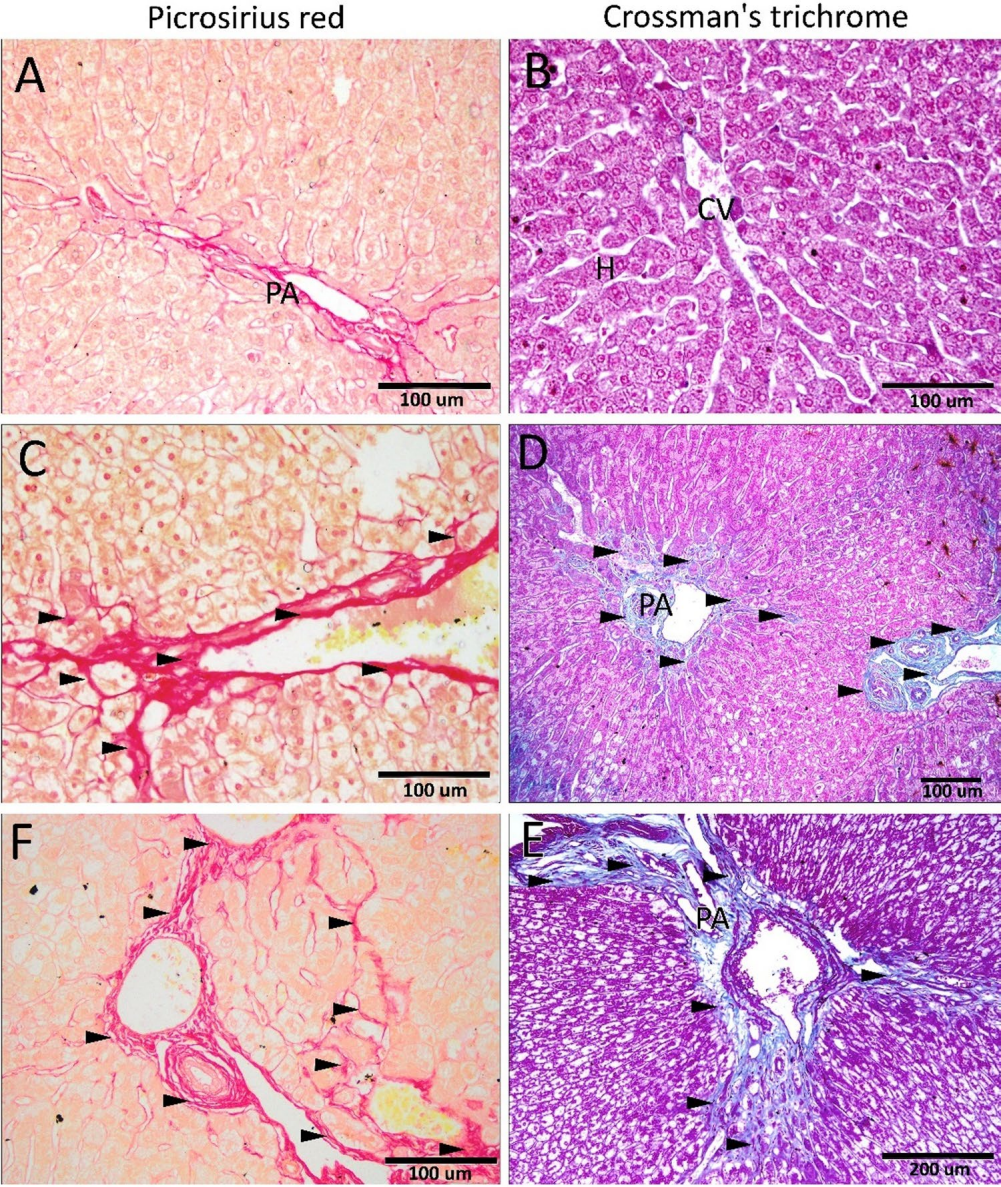
The collagen content and the fibrous tissue within the liver tissue. The Picrosirius red (left column) and Crossman’s trichrome (right column) showed (**A, B**) normal amounts of collagen fiber in the **c**ontrol groups. (**C-F**) Rabbits fed on different concentrations (5, 10 & 15 gm/kg) of neem leaves showed an abnormal increase in the collagen fibers (arrowheads) in the portal area, which extended beyond the portal area. PA portal area, H hepatocytes, CV central vein.

**Fig. 6:**
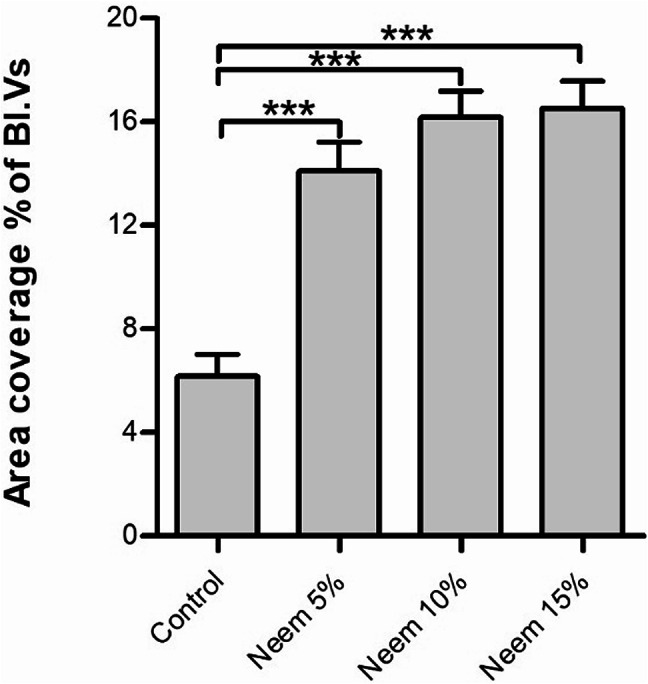
quantification of the coverage area percentage of the collagen fibers in the control and after supplementation of 3 different concentrations of neem (5, 10, 15%).

Picrosirius red and Crossman’s trichrome were used to demonstrate the fibrous tissue and the collagen content within the liver tissue (Fig. 5). Control groups showed normal amount and distribution of collagen fiber and fibrous tissue, mainly within the portal area (Fig. 5A, B). Rabbits fed on neem leaves showed an abnormal increase in the deposition of collagen fibers and fibrous tissue in the portal area. Moreover, these fibers extended beyond the portal area between the adjacent hepatic sinusoids (Fig. 5C-F). Upon quantification of the coverage area percentage of the collagen fibers, the increase of collagen in the case of the three concentrations was highly significant (*P* < 0.001), compared to the control (Fig. 6).

## Discussion

In our experiment, we investigated the effect of administration of three different concentrations of neem leaves on the growth and biochemical parameters in rabbits, focusing on the histological examination of the liver, which constitutes a major organ responsible for metabolism.

The results showed an insignificant decrease in body weight gain in the neem-fed groups in all the examined ages, compared to the control group similar to previous work on leaf powder in male rabbits^24^ and female rabbits^22^and also in the case of leaf powder in guinea fowl^23^ and leaf extract in broiler^21–24^. The reduction in body weight gain is accompanied by an insignificant decreased feed intake (FI) leaf powder in female rabbits^22^ which consequently led to an insignificant increase in the feed conversion ratio (FCR). This agrees with previous research, where the supplementation of neem leaves at 5% to rabbits’ diets^21^ did not show any significant difference in FCR. It has been reported that supplementation of neem leaf oil to rabbits’ diets significantly decreased FI^25^ however, unlike ours, they found a decreased FCR. Unlikely, some researchers found that supplementation of neem leaves to rabbits’ diets improved the BW and FI in rabbits^26^ and broiler^27^ after supplementation with leaf powder^26,27^.

The results revealed a significant decrease in the rabbit’s cecum weight in the two higher neem concentrations. This could explain the reduction in live weight, carcass weight and abdominal fat weight because the cecum is very important in the digestion of feed to highly nutritive elements in rabbits^28^. The cecum of rabbits makes them benefit from the complex carbohydrates of plants, where microbial fermentation of indigestible lignin and cellulose by abundant microorganisms into the produced short-chain fatty acids. The gut microbiota was implicated in the regulation of many physiological processes, including digestion, neuroendocrine, immune response and even normal behavior of rabbits^29^. Since neem is known for its bactericidal effect, it might manipulate the cecal microbiota, reducing their activity and shrinking the cecum size^15^.

The head and liver weight increased in the three concentrations of neem leaves^25^. The heart weight increased significantly, as well in the lowest concentration. It has been reported that different concentrations of neem leaves led to variable effects on some organs, such as the liver and kidney^23^. The increase in kidney weight could suggest a potential chemically induced subacute or chronic renal toxicity^30^.

Biochemical blood parameters are usually used as indicators for the physiological, nutritional, and pathological status of animals, and have the potential to be used to elucidate the impact of nutritional factors and additives supplied in diet^31^. The current study showed that supplementation of different levels of neem leaves to rabbits’ diets did not show significant changes in biochemical blood parameters, such as total protein and glucose level^23^; serum AST, ALT, creatinine concentration^32,33^ and urea concentration^32^compared to the control group. Moreover, the only parameter that significantly (*P* < 0.05) increased at 5 and 10% was the high-density lipoprotein (HDL)^34^however, the increase in the highest dose was insignificant.

The current study revealed a slight increase in total protein at 10%, while a slight decrease at the 5 and 15% concentration. This occurred due to the extra decrease in the globulin at the 5 and 15% concentrations and the nearly equal slight increase in albumin concentration in all groups. However, a significant increase of albumin at 10 and 15% of neem leaves was reported before^23^. These changes could indicate an impact on the immune system or protein metabolism, suggesting that neem may influence immune function or alter protein synthesis in the body. Low levels of globulin might indicate antibody deficiencies, meaning a weakened immune system^35^. Low globulin levels can also occur due to malnutrition caused by eating an imbalanced diet. Further investigation would be needed to fully understand the implications of these changes in globulin levels.

In the current study, cholesterol concentration insignificantly increased after neem leaf supplementation, especially in the lowest dose. This observation contradicted the findings of two previous research, either after the supplementation of neem leaves^33^ or crude neem leaf extract^36^ to rabbits. Both scientific groups reported a significant (*P* < 0.05) decrease in cholesterol.

In the current study, the glucose concentration did not change significantly after leaf supplementation. A previous study reported that neem leaves at 10 and 15% reduced glucose concentration significantly (*P* < 0.05), however, the reverse occurred at 5% ^23^. In the current study, the urea concentration decreased significantly after neem leaf supplementation in the highest dose. This finding agrees with a previous study, where a significant (*P* < 0.05) decreased urea concentration even at lower doses (5, 10 and 15%)^33^. Possible mechanisms behind the decrease in blood urea levels at higher neem concentrations can reflect protein metabolism and kidney function. The decrease in blood urea, coupled with the increased kidneys’ weight, might reflect an attenuated kidney function^35^. However, it was not dose-dependent and needs more study.

In the current study, both serum AST and ALT concentrations insignificantly increased after neem leaf supplementation. Increased serum AST and ALT concentration might be explained by the loss of membrane integrity of hepatic cells, which allows for the leakage of enzymes from the cells, raising their levels in the serum^37^. However, it has been reported an insignificant reduced serum AST and ALT in rabbits fed neem leaf meal at 10 and 15% ^33^, whereas at 5% (NLM) increased serum AST and ALT, compared to the control group.

The results of this study and the previously discussed controversial variations, which were not proportional or did not follow a consistent rule, encouraged our group to analyze the histological structure of the liver to confirm whether the neem is beneficial or detrimental to rabbits. The microscopic examination of the liver sections from the control rabbit group revealed a normal histological structure of the hepatic lobules. The livers of rabbits which fed on neem leaves of different concentrations for 10 weeks revealed some degenerative changes, such as hydropic/vacuolar degeneration of hepatocytes, angiopathic changes such as congestion of both central vein and portal vein, oedema in the portal area and diffused periportal leucocytic infiltration. This explains the increased weight of the liver. Similar lesions were reported in hepatotoxicity caused by carbon tetrachloride (CCL4)^38^ and Methotrexate^39^ in rats. The signs of hepatotoxicity could be explained by lipid peroxidation and the inhibition of oxidative enzymes, which result in the accumulation of the free radicles that damage the liver in rats^40^.

The glycogen granules in the hepatocytes cytoplasm of the control group appeared normal due to the strong magenta reaction with PAS staining. The glycogen content was depleted in most hepatic lobules in rabbits fed on lower neem concentrations and the severity increased proportionally with the amount of neem leaves. This indicates less glycogen production by negatively affected or intoxicated hepatocytes^41^. Of note is that the affected liver of neem-fed rabbits resulted in either reduced glycogen synthesis or enhanced glycogen catabolism (glycogenolysis). In this study, although the liver was affected, reduced in weight, and its glycogen was depleted, it seems plausible that dysfunctional glucose metabolism is balanced out by utilizing alternative sources other than glucose because the blood glucose level did not change significantly, which could increase glucose disposal into the circulation^42^ in mice, which needs more biochemical study to add to diabetic research.

The abnormal increase in the deposition of collagen fibers in the portal area and extended between the adjacent hepatic sinusoids in the case of rabbits fed on neem leaves, compared to the control group, indicates a harmful effect of neem on the liver^43^ similar to that reported after thioacetamide-induced liver cirrhosis in rats. We assume that the increase in the deposition of the fibrous tissue is a result of the death and degeneration of hepatocytes, which are replaced by this connective tissue^44,45^ in rabbits^46^ and rats^44–47^. A complex interaction among cells occurs during fibrogenesis in the liver, where intoxicated hepatocytes release reactive oxygen species and undergo apoptosis. This induces inflammatory cells and white blood cell recruitment. Inflammatory cells activate hepatic stellate cells to secrete collagen and accumulation of extracellular matrix proteins. The activated inflammatory and stellate cells secrete inflammatory chemokines, express cell adhesion molecules, and a circle in which inflammatory and fibrogenic cells stimulate each other^48^. The accumulation of extracellular matrix proteins distorts the hepatic architecture by forming a fibrous scar. Consequently, cirrhosis leads to hepatocellular dysfunction, hepatic insufficiency, increases intrahepatic resistance to blood flow and portal hypertension^49^.

The controversial data of neem leaf studies might be attributed to the variability in neem leaf composition, which has been reported in India^50^ and Malaysia and Sudan^51^. They concluded that there are individual genetic differences among neem trees, and synthesis of different compounds, which is not dependent upon temperature, humidity or rainfall. Therefore, a systematic study for tree improvement with a population of mother trees with desired traits is essential^50^.

*In conclusion*, the supplementation of neem leaves to rabbits decreased feed intake, body weight, carcass weight and cecum weight. Moreover, neem leaves induced an increase in serum AST and ALT concentration, together with the degenerative changes of hepatocytes, angiopathic changes and fibrosis. In light of the above findings, we can conclude that administration of neem leaves for 10 weeks induced a dose-dependent hepatotoxicity in the liver of rabbits. Therefore, we suggest avoiding adding high doses of neem leaves to the rabbit ration. Further research should be carried out to study which animals can benefit from the neem leaves or are sensitive to their use.

## Data Availability

The data that support the findings of this study are available from the corresponding author upon reasonable request.
